# Intelligent Complementary Multi-Modal Fusion for Anomaly Surveillance and Security System

**DOI:** 10.3390/s23229214

**Published:** 2023-11-16

**Authors:** Jae-hyeok Jeong, Hwan-hee Jung, Yong-hoon Choi, Seong-hee Park, Min-suk Kim

**Affiliations:** 1 Department of Electronic Information System Engineering, Sangmyung University, Cheonan 31066, Republic of Korea; 2023d1013@sangmyung.kr; 2 Department of Human Intelligence and Robot Engineering, Sangmyung University, Cheonan 31066, Republic of Korea; hwanyo14@gmail.com (H.-h.J.); choiyonghoon1103@gmail.com (Y.-h.C.); 3 Intelligent Convergence Research Laboratory, Electronics and Telecommunications Research Institute, Daejeon 34129, Republic of Korea; pshee@etri.re.kr

**Keywords:** surveillance and security, multi-modal, anomaly detection, 3D convolutional autoencoder, anomaly classification, slowfast, GTA dataset

## Abstract

Recently, security monitoring facilities have mainly adopted artificial intelligence (AI) technology to provide both increased security and improved performance. However, there are technical challenges in the pursuit of elevating system performance, automation, and security efficiency. In this paper, we proposed intelligent anomaly detection and classification based on deep learning (DL) using multi-modal fusion. To verify the method, we combined two DL-based schemes, such as (i) the 3D Convolutional AutoEncoder (3D-AE) for anomaly detection and (ii) the SlowFast neural network for anomaly classification. The 3D-AE can detect occurrence points of abnormal events and generate regions of interest (ROI) by the points. The SlowFast model can classify abnormal events using the ROI. These multi-modal approaches can complement weaknesses and leverage strengths in the existing security system. To enhance anomaly learning effectiveness, we also attempted to create a new dataset using the virtual environment in Grand Theft Auto 5 (GTA5). The dataset consists of 400 abnormal-state data and 78 normal-state data with clip sizes in the 8–20 s range. Virtual data collection can also supplement the original dataset, as replicating abnormal states in the real world is challenging. Consequently, the proposed method can achieve a classification accuracy of 85%, which is higher compared to the 77.5% accuracy achieved when only employing the single classification model. Furthermore, we validated the trained model with the GTA dataset by using a real-world assault class dataset, consisting of 1300 instances that we reproduced. As a result, 1100 data as the assault were classified and achieved 83.5% accuracy. This also shows that the proposed method can provide high performance in real-world environments.

## 1. Introduction

Owing to the limitations of the real-time manual monitoring closed-circuit television (CCTV) system, an intelligent autonomous monitoring system capable of automatically detecting abnormal states (behaviors) is required. Traditional intelligent CCTVs have primarily been anchored in sensor-based and vision processes. However, with the improvement in the overall hardware performance and advanced artificial intelligence (AI), many prior works are focusing on automatically detecting abnormal states using deep learning (DL) techniques [1].

Most of the AI-based CCTV based on the DL method is normally divided into phases: (1) the video anomaly detection (VAD) system and (2) video anomaly classification (VAC) system. VAD is typically applied to unsupervised learning methods. The method can adopt the one-class classification (OCC) system that learns the features of normal events and it can also distinguish abnormal events depending on the learned normal features. In contrast, VAC is based on trained supervised learning methods. This has a multi-class classification to learn the features of predefined abnormal events and it can classify input events into one of the predefined classes. The two different learning methods are difficult to simultaneously apply in the development and implementation of an end-to-end system, so surveillance security systems have adopted only one single method.

However, there are inherent issues with relying solely on one scheme (VAD or VAC). (i) The VAD system is based on the OCC method, making it challenging to classify specific abnormal events. However, a clear classification of abnormalities is essential to respond to abnormal events in real time. Ultimately, this leads to the need for supervisors to monitor CCTV systems in traditional manual surveillance systems. (ii) The VAC system typically deals with the features of normal events during the learning process. However, as the range of the normal state is wide, it is also difficult to learn all classes of the normal event features [2]. (iii) Even when normal event learning problems are resolved, the VAC system still faces challenges in detecting anomalies within the crowd. In a crowd, various normal and abnormal events occur simultaneously, so object-level classification is required. Nevertheless, unlike image-based data, video-based data and model characteristics pose challenges for real-time monitoring systems due to their substantial computational demands.

**Figure 1:**
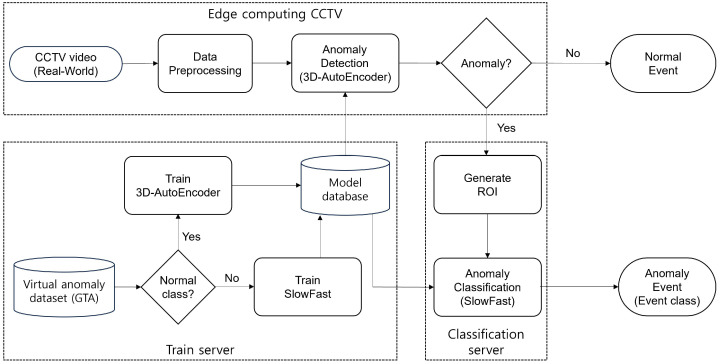
Structural flowchart of anomaly surveillance and security system for multi-modal fusion.

In this paper, we propose an intelligent multi-modal surveillance system that incorporates both VAD and VAC to address existing challenges. As shown in Figure 1 (flowchart of the proposed structure), VAD only detects abnormal states, activating VAC only when anomalies are identified in real-time monitoring scenarios. By integrating both VAD and VAC, this structure tackles the anomaly classification problem in the traditional single VAD system, and it can similarly address the problem of defining normal events in VAC. In addition, for enhanced classification performance, we utilize the VAD’s output feature to VAC’s input. VAD is implemented to adapt the 3D AutoEncoder (3D-AE) model, comparing the errors between the actual and reconstructed frames to pinpoint the precise anomaly locations. For VAC, we adopt the SlowFast neural networks [3,4] based on a 3D convolution neural network (3D-CNN). This approach enhances accuracy by using precise ground-truth tube input data derived from VAD, eliminating the necessity for separate detection models, such as YOLO [5], etc. This also leads to reduced computational costs for object detection and system performance, making it a more favorable option compared to other similar VAC methods.

Moreover, we focused on the challenge of utilizing the existing open datasets for benchmarking model performance in the context of a real-world anomaly detection system. In the case of open datasets designed for anomaly detection, they have a limited range of anomaly patterns and do not accurately capture the diverse illumination conditions found in real-world environments. Learning under limited illumination conditions does not completely represent the real surveillance system environment because the data distribution varies based on illumination [6]. Moreover, as anomaly events occur with low probability, there are quantitative limitations when it comes to gathering real-world data and imitations due to issues, such as risk and stability.

To overcome the problem, we constructed a dataset using a virtual environment. The new virtual environment dataset is produced using the Grand Theft Auto 5 (GTA5) gaming framework that is useful in reproducing assault, intrusion, gun accidents, and explosion accidents data. The dataset has a wider variety of background and limitations factors than the traditional open datasets, and it can give a highly effective performance in detecting anomaly events in real scenarios.

## 2. Related Works

Previously, surveillance monitoring of hazardous facilities was initially conducted manually to enhance efficiency and reliability due to time constraints. However, there has been a significant endeavor to develop autonomous monitoring systems for security and hazard facilities. Before the development of the AI model, an automated monitoring system that utilized image processing techniques with sensor-based CCTV cameras was primarily employed. In particular, an automated monitoring system was used to detect abnormal states, such as background removal, and to deliver dangerous signals to managers using detection sensors [7,8]. However, these methods needed to require human resources, and it is still difficult to implement fully automated surveillance security systems. Thus, after the development of AI-based methods, many attempts have occurred to implement a fully automated surveillance system.

### 2.1. Unsupervised Learning-Based Video Anomaly Detection

As normal states occur more frequently than abnormal states, the normal states are diverse and complex. Therefore, it is practically hard to learn all the normal states for abnormal detection using multi-class classification. To solve the problem, many of the prior works focused on using OCC to learn normal-state data. With the increase in CNNs in the field of computer vision, unsupervised learning-based anomaly detection was conducted [9], and video-based anomaly detection works with generative models, such as AutoEncoder and the generative adversarial network (GAN), were attempted [10,11]. Generative models are fundamentally utilized with the learning of normal features to reconstruct normal frames with a high probability of occurrence. It can perform anomaly detection compared to abnormal frames with a low probability of occurrence. This shows high performance under unsupervised learning-based VAD. Recently, many prior works were conducted and incorporated the classical clustering methods, such as K-means and support vector machine (SVM), into AutoEncoder [12,13,14]. These methods use latent features extracted from Encoder as the clustering to detect outliers that do not fall within the normal category. The models are also evaluated at a similar or higher level to the performance of the VAD model. However, the multi-class classification problem still existed in a method based on OCC in the prior works. In this paper, we attempt to combine VAD and VAC to address multi-class classification. VAD is primarily used to detect abnormal events and generate an ROI for event occurrence points. In addition, by using the 3D-AE model [15], the frame reconstruction error value is expressed in pixels so that the ROI that will be used as input data for VAC can be easily created.

### 2.2. Supervised Learning-Based Video Anomaly Classification

Multiple classifications of anomaly detection schemes are necessary. Consequently, supervised learning-based methods require datasets for the learning process to construct each anomaly state class and its associated features. Previously, for data processing such as CCTV video, the video data were divided into frames, and a CNN-based object detection algorithm using 2D images for high-performing detection models is shown in [16,17]. However, because the 2D-CNN-based scheme can only learn the spatial features of the image, it does not capture the temporal features inherent in video data. To address this problem, the prior related works tried to combine models, such as 2D-CNN and the Recurrent Neural Network (RNN), to learn the temporal features of video data [18,19]. This led to improved video recognition capabilities; however, handling long-term video data remained challenging due to the long-term dependencies of RNNs. Afterward, the surveillance system for high performance applied 3D convolution that can learn the time axis of video data by extending the 2D convolution based on one dimension. As a result, the advanced research on video data recognition and surveillance systems using 3D-CNN has been continuously increasing and improving. Recently, a vision model such as Vision Transformer (ViT) [20] applied by a transformer gave a high performance in the fields of Natural Language Processing (NLP) for the achievement of the state of the art (SOTA) by using the vision task. However, the transformer model should be required for large-scale pre-training data and its large capacity, so it is also difficult to apply in the current computing system. Additionally, the previous work focused on only the classification and performance evaluation of predefined event classes. However, it is still a problem in that there is no scenario for the normal events of the input data in the VAC system. Therefore, in this paper, we adopted multi-modal fusion with VAC and VAD to classify the abnormal events throughout the whole learning process. For classification, we chose the SlowFast neural network, which continues to be widely used in various domains of action recognition and is applied to surveillance systems [21].

## 3. Convergent Analysis and Preprocessing

**Figure 2:**
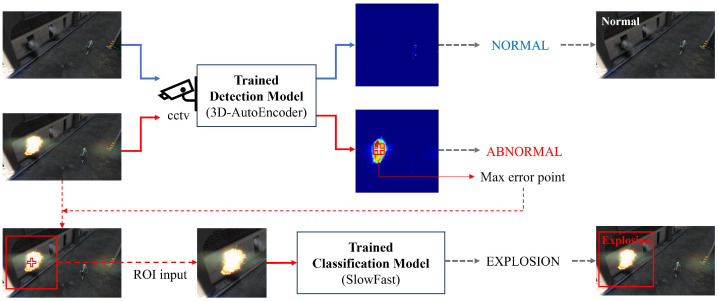
Multi-modal anomaly surveillance and security workflow.

In this paper, an intelligent monitoring surveillance system based on multi-modal methods is implemented to distinguish between two events, namely, learning the features of normal and abnormal events. Figure 2 shows the structure proposed by an intelligent CCTV security system. The raw data are collected through CCTV in a computing real-time system, and they can be delivered to the 3D-AE, which is a pre-trained anomaly detection model for data preprocessing. It can distinguish the normal state from the abnormal state. When an abnormal state is detected, the corresponding data are sent to the classification model trained using SlowFast, allowing for the specific classification of the abnormal state.

As shown in Figure 2, the multi-modal-based intelligent monitoring system primarily detects abnormal events based on the 3D-AE model. Following the initial detection, the detected anomalies are further classified using SlowFast, which is a classification model based on a 3D-CNN backbone. It can classify more specific anomaly events using red–green–blue (RGB) input. The abnormal detection (3D-AE) can reconstruct the damaged anomaly frame, and it also achieves pixel value errors to be compared with the original frame for an abnormal-state occurrence. The pixel value has errors in the appearance of the reconstructed frame. Using this approach, the frame and location of abnormal-state occurrences are identified within the SlowFast model. To achieve a more detailed classification, an ROI is generated around the point of the anomaly occurrence. The SlowFast model can then be divided into a slow path that is sensitive to spatial features and a fast path that is sensitive to temporal features. It can also enable a high feature extraction and classification level for RGB-based ROI inputs.

The contributions in this paper are as follows: (i) A proposed method for an efficient multi-modal fusion surveillance system that combines both VAD (unsupervised learning) and VAC (supervised learning). (ii) Offering a solution for learning normal states and enhancing the computational efficiency of pre-detecting anomaly states. (iii) Improvement in the learning accuracy in the VAC and complementary system performance using a reconstruction-based VAD to enable ROI generation. (iv) More specific and correct abnormal classification using multi-modal fusion. (v) Creating a self-produced GTA (virtual video data) dataset to facilitate data collection from a broader range of backgrounds, thus enhancing the efficiency of the learning process.

### 3.1. Dataset

**Figure 3:**
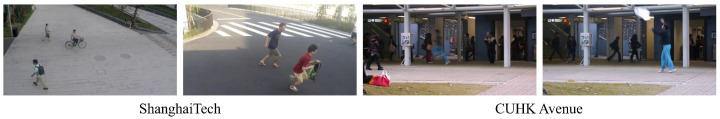
Open datasets for anomaly detection.

In general, a CCTV-based video dataset for abnormal states (behavior) is required for abnormal detection and classification work in secure facilities. To implement the proposed method, the dataset needs to be used for both the detection and classification models concurrently. Furthermore, the dataset should include a specific proportion of normal and abnormal states, covering various classes of abnormal states, including assault, intrusion, firearm accidents, and explosion accidents. However, in usual behaviors, the datasets for detection and classification are distinct, making it challenging to simultaneously train both models with open datasets. The datasets such as CUHK Avenue [22] and ShanghaiTech [23] have been primarily used in the one-class classification of anomaly detection tasks. These are the street videos taken from the same camera angle as the CCTV: the training dataset with normal states and the test dataset for the abnormal states. The normal states are usually classified as pedestrians walking, and abnormal states are expressed as running, riding a bicycle, and driving a car. While these datasets are frequently employed for anomaly detection tasks, utilizing them to classify abnormal states within the context of security facilities remains challenging. Specifically, the dataset mentioned above does not encompass the abnormal states that need to be detected in security scenarios, such as assault, intrusion, firearm incidents, and explosion events. Furthermore, as shown in Figure 3, this is the case of the Chinese University of Hong Kong Avenue dataset (CUHK Avenue) and it is rather monotonous because the camera is fixed at a single point in time. Therefore, it is also difficult to apply them to changing real-world situations because both datasets were taken in a constant illumination environment. In addition, it is necessary to collect datasets containing crimes and accidents for proper classification work. Typically, the University of Central Florida 101 dataset (UCF-101) [24] and Human Motion Database 51 dataset (HMDB-51) [25] are frequently used in video classification for only actions, such as sports and playing musical instruments; thus, there are problems to apply. Furthermore, there is an issue related to the learning of abnormal states. Each video does not contain normal states, which poses a challenge in the learning process.

### 3.2. Data Generation and Supplement

To collect data for our research, we utilize a game virtual environment that closely simulates the real world. There are precedents [26,27] in the previous works, where experiments have been conducted by collecting datasets using a similar game environment. Open-world games, such as Grand Theft Auto (GTA), are suitable for reproducing realistic scenes owing to their excellent realism and good graphics. Additionally, because abnormal conditions can be simulated using various tools within a virtual environment, datasets involving criminal or threatening behaviors, which are challenging to replicate in the real world, can be efficiently and safely generated.

**Figure 4:**
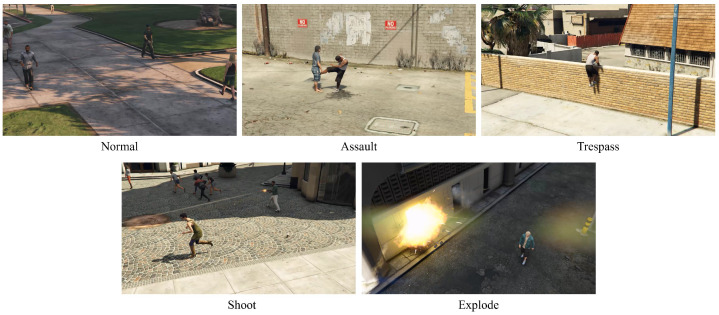
Data generation sample using Grand Theft Auto V.

As shown in Figure 4, the datasets can be collected in the GTA5 virtual gaming environment by setting indoor and outdoor locations, and they are similar to datasets in CCTV. The normal state for the detection model comprises videos depicting pedestrians walking normally on the street, and a total of 78 such videos were collected. The abnormal states to be shared in the detection and classification models are shown in Figure 4. Abnormal-state classes are defined in each environment as follows: detection of intrusion in general facilities, assault in public places, and explosions in hazardous facilities. We define the above reference into four classes, (a) assault, (b) intrusion, (c) gun accident, and (d) explosion, consisting of 92, 100, 108, and 100 datasets, respectively.

**Figure 5:**
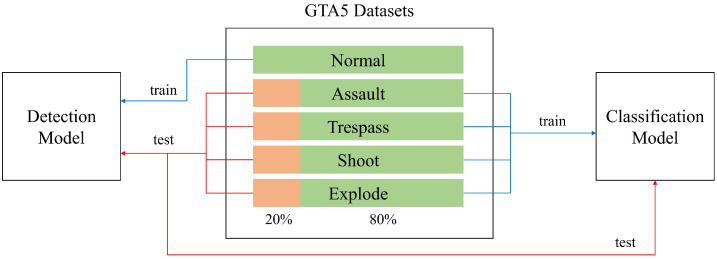
Data process flow for multiple deep learning models.

For the abnormal state, as shown in Figure 5, 20 randomly selected datasets for each class can be used for the test verification of the two models, and the remaining images are composed of the learning datasets of the classification models. Each dataset is approximately 8 to 20 s in duration and consists of RGB-color channel videos with a resolution of 1440 × 830 pixels and a frame rate of 30 fps.

### 3.3. Data Preprocessing Module

Learning performance can be evaluated using area-under-the-curve (AUC) scores as an indicator of performance for anomaly detection performed using unsupervised learning-based detection models. The area (integral value) of the receiver-operating-characteristic (ROC) curve drawn by calculating the true positive rate (TPR) and false positive rate (FPR) becomes the AUC score; the ground truth is required to calculate the TPR and FPR. In other words, a ground truth for an abnormal-state image used to evaluate the detection model should be generated. The normal-state frame of pedestrians walking in the video stream is labeled as 0, and the abnormal-state frames such as an assault, an intrusion, a gun accident, and an explosion accident are labeled as 1. The learning model using the ground truth and prediction results can be evaluated by the AUC.

**Figure 6:**
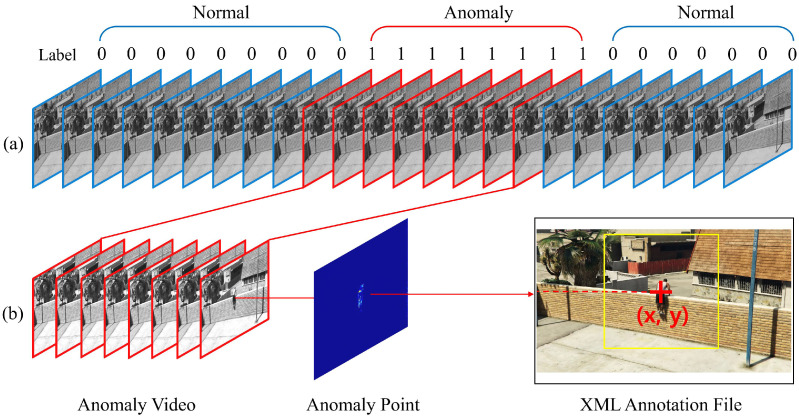
(**a**) Labeling frames for detection model, and (**b**) XML annotation file and anomaly video for classification model.

The dataset for supervised learning-based classification can create an annotation file defined by the start and endpoints of the abnormal state that includes the coordinates of the abnormal-state occurrence point. As for the data preprocessing, 64 frames included with anomalous frames can be extracted and resized at a fixed rate of random size between 256 and 320, based on the short axis of the frame. After that, as shown in Figure 6, the occurrence points of the abnormal states need to be set as the center coordinate and cut into a 224 × 224 size.

## 4. System Materials and Methods

### 4.1. Anomaly Detection

**Figure 7:**
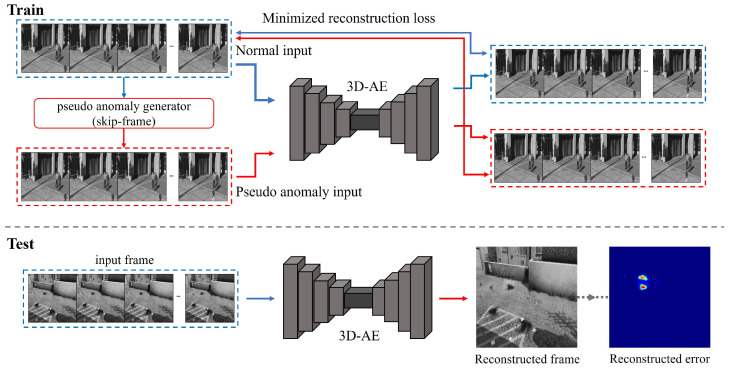
3D-AE workflow with skip-frame methodology.

In this paper, VAD using 3D-AE is conducted and the method is extended with a skip-frame approach [15]. The skip frame is one of the methods widely used in recent prior works, and it is used to improve the problem [28,29,30] of restoring abnormal frames via AutoEncoder. Figure 7 shows the overall structure of the 3D-AE using the skip-frame method. The AutoEncoder can only learn to reconstruct a normal frame that does not accurately reflect an abnormal state. The reconstructed abnormal frame has noises and damages at the point of anomaly. The high pixel value errors can be obtained to be compared with the original frame.

#### 4.1.1. Three-Dimensional Convolutional AutoEncoder

**Figure 8:**
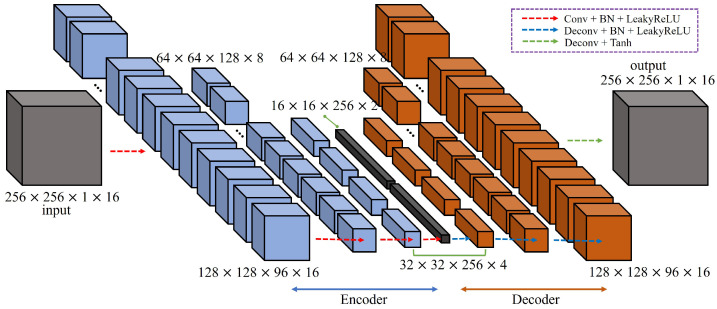
3D-AE structure.

In this paper, the 3D-AE is used for an anomaly detection model based on future frame prediction. Collecting comprehensive normal data is challenging because the categories of the normal states are so diverse that it is impossible to gather data representing all possible normal scenarios. Therefore, it is more effective to learn the categories of the normal states through unsupervised models in advance rather than to learn the multiple classifications including the normal classes through supervised learning. The unsupervised model, adapted with AutoEncoder using 3D convolution, can learn and classify spatiotemporal features. The structure of the model is composed of four 3D convolution layers in the Encoder and Decoder, respectively, and the structure is shown in Figure 8.

#### 4.1.2. Skip-Frame Methodology

Recently, it has been highlighted that AutoEncoder can reconstruct and perform incorrect results [28,29,30], and several methods have been introduced to limit the reconstruction thresholds to solve this problem [31]. The previously mentioned issue is tackled through the skip-frame method, which simulates abnormal data with irregular rates by skipping specific frames and training the model to utilize them as an indicator to set reconstruction thresholds. The skip-frame method generates pseudo-abnormal data by skipping several frames of a specific value and learning it with normal frames at the same time. Through this, by limiting the reconstruction threshold, it is induced to reconstruct only the normal data of the test dataset.

### 4.2. Anomaly Classification

**Figure 9:**
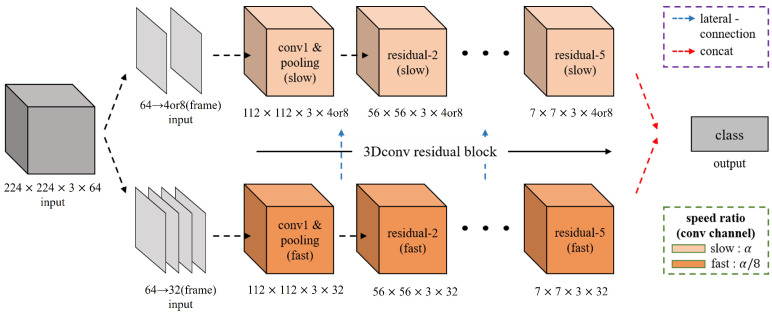
SlowFast neural network structure.

We propose the classification method using the SlowFast neural network to classify video-based anomalies. It is a 3D ResNet-based classification that classifies video data by receiving 64 consecutive frames of size 224 × 224. The structure in Figure 9 is divided into the slow pathway and the fast pathway. The slow pathway has 4 or 8 frames separated at regular intervals from a total of 64 frames, along with multiple convolutional channels, to extract the contextual and spatial features, including the background of the video data. The fast pathway uses 32 frames out of 64 frames and a small number of convolution channels to extract the motion and temporal features of the video data. The two separate output maps are equalized in size through lateral connections to enable sharing, and they are utilized for classifying the input video data based on the extracted features.

## 5. Experimental Results

### 5.1. Experimental Setup

In this paper, we set the hyper-parameters for 3D-AE, such as the batch size = 4, learning rate = 0.0001, epoch = 60, and skip-frame parameters = 2, 3, 4, and 5. Additionally, SlowFast is a set of parameters as follows: batch size = 16, learning rate = 0.001, and epoch = 50.

### 5.2. Evaluation of Anomaly Detection Models

The ROC curve is set as an evaluation index and reconstructs the prediction frame through the enhanced 3D AutoEncoder. In addition, it compares the reconstructed frame and the ground-truth frame to derive an anomaly score using the peak-signal-to-noise ratio (PSNR). The PSNR is obtained as shown in Equation (1) using the mean squared error (MSE). The receiver operating characteristic:

(1) $$\mathrm{MSE}=\frac{1}{M\times N}\sum_{m=1}^{M}\sum_{n=1}^{N}{\left[I_{1}\left(m,n\right)-I_{2}\left(m,n\right)\right]}^{2}$$

*M* and *N* denote the number of matrix pixels of the input frames $I_{1}$ and $I_{2}$, respectively. Using the derived MSE, the PSNR of the reconstruction frame and ground-truth frame are obtained.

(2) $$\mathrm{PSNR}=10\log_{10}\left(\frac{R^{2}}{\mathrm{MSE}}\right)$$

In Equation (2), *R* denotes the maximum possible pixel value of the frame of the input. Finally, we derive the anomaly score *S* using the minimum–maximum normalization. This process is shown in Equation (3).

(3) $$S=1-\frac{\mathrm{PSNR}-\min(\mathrm{PSNR})}{\max(\mathrm{PSNR})-\min(\mathrm{PSNR})}$$

**Figure 10:**
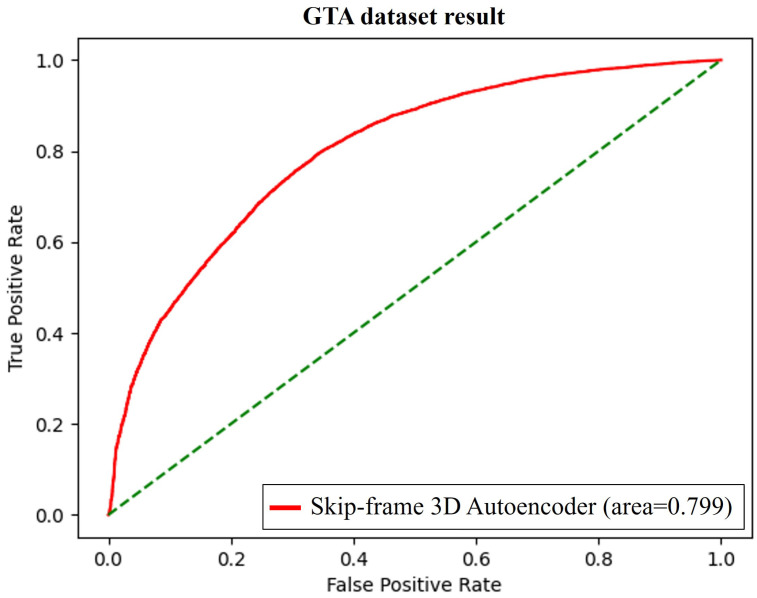
ROC curve and AUC score of GTA dataset.

In this paper, we use the indexes to evaluate a GTA dataset-based learning model, and it can additionally compare other learning models using open datasets, such as CUHK Avenue and ShanghaiTech. All the data frames were resized with an image of size 256 × 256 in grayscale, and the result is shown in Figure 10 and Table 1. The learning model based on the GTA dataset showed an approximately 4% higher detection performance than the ShanghaiTech-based model, despite having a complex environment level similar to ShanghaiTech. Even though the accuracy is approximately 4.72% lower than that of the model using the Avenue dataset, the AutoEncoder scheme can make an increase in the reconstruction threshold value owing to large environmental changes, such as the amount of illumination. Thus, the GTA dataset also has a variety of compositions and illuminations compared to that of Avenue, making it slightly low in accuracy.

**Figure 11:**
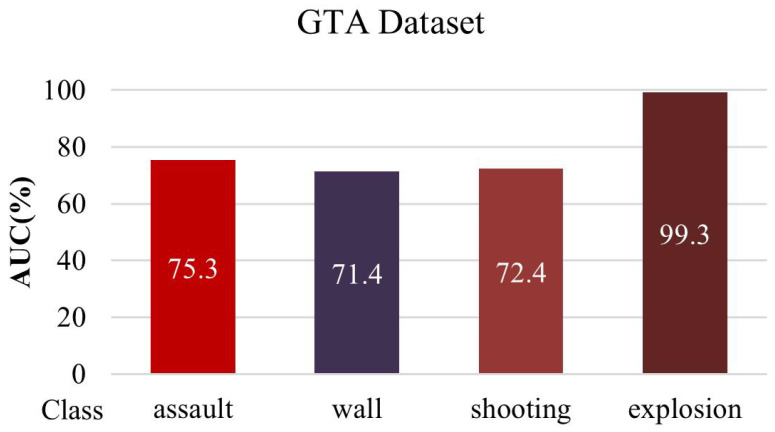
AUC score for each class.

**Figure 12:**
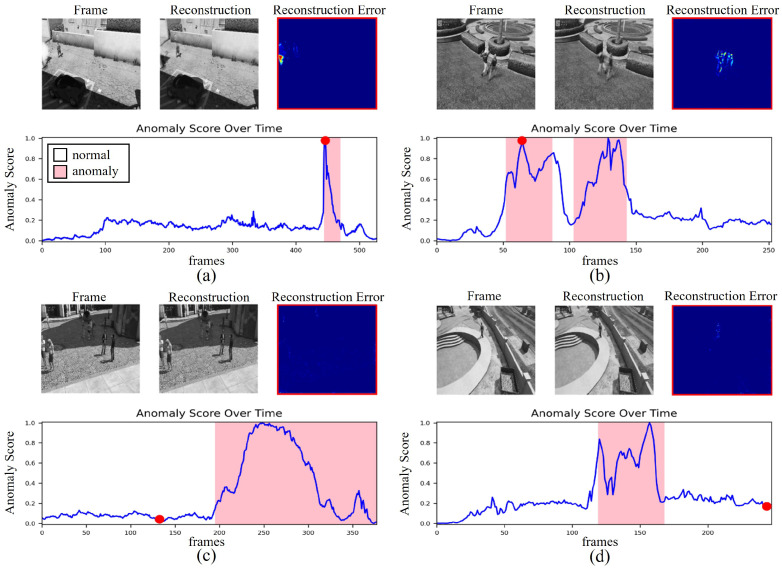
Experimental results based on anomaly score: classes of (**a**) explosion, (**b**) assault, (**c**) shooting, and (**d**) trespass.

Figure 11 shows the AUC and anomaly scores for each class. It can be seen that the explosion and assault classes, which are generally temporary or continuous abnormal states, show relatively better performance. Figure 12 shows that the anomaly score represents the areas involved with the abnormalities in (a) and (b). The anomaly scores in (c) and (d) are low in the normal-state areas. Furthermore, pixel value errors are generated when an abnormal event occurs in the reconstruction error frame. As a result, the reconstruction-based 3D-AE can have the result of a proper ROI.

### 5.3. Evaluation of Anomaly Classification Models with 3D-AE Preprocessing

**Figure 13:**
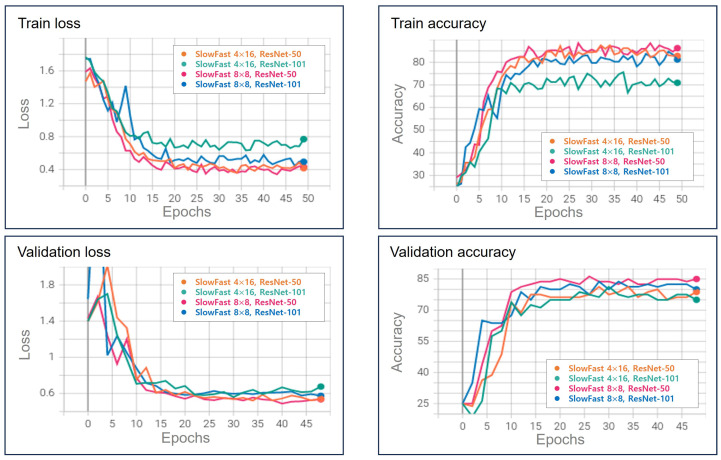
Experimental results of SlowFast models using GTA datasets (our dataset).

We assess the anomaly classification performance of the multi-modal system using a SlowFast model based on 3D-AE. In addition, classification performance comparisons are made with an open dataset. As listed in Table 2, 4 × 16 means that the frames used in the slow pathway have 4 of the 64 frames for the input size, and 8 × 8 means that 8 frames are designated for the slow pathway. The box crop preprocessing approach is adapted to extract regions of 224 × 224 pixels based on the anomaly-centric coordinates. This technique aligns with the spatial location of the maximum error value in the reconstruction error obtained from the 3D-AE model. On the other hand, random crop implies learning by randomly sectioning a portion of the frame to a size of 224 × 224. The training process is executed over 50 epochs, and the learning and validation outcomes, particularly those associated with the GTA dataset and ‘Box crop’ preprocessing, are shown in Figure 13.

**Figure 14:**
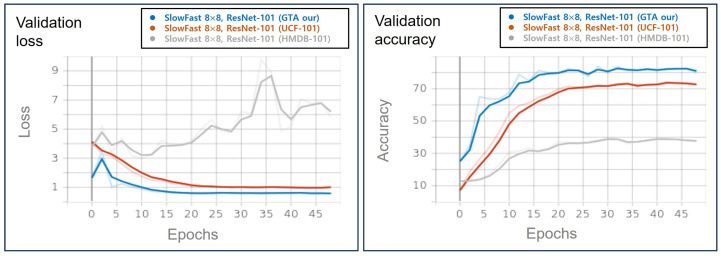
Experimental results of GTA (our dataset) and UCF-101 and HMDB-51 datasets.

Figure 14 shows the validation results of the UCF-101, HMDB-51, and GTA datasets of SlowFast 8 × 8 ResNet-101. The accuracy is approximately 7W2.31% for UCF-101 and 37.5% for HMDB-51, and the results were approximately 80% on the GTA dataset. UCF-101 and HMDB-51 are frequently used as performance indicators for video classification models. It can be confirmed that the GTA dataset is an abnormal-states classification dataset with the identification approximately 5% higher than the verification accuracy of UCF-101 and 30% or more compared to HMDB-51.

**Figure 15:**
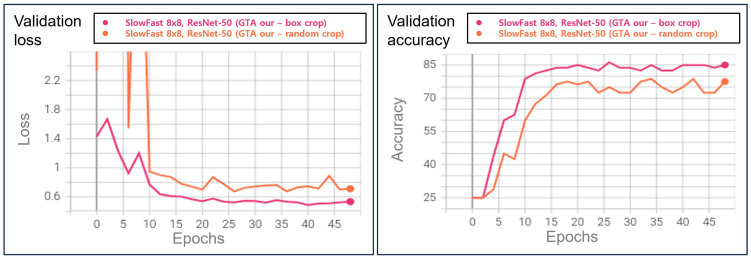
Experimental results of preprocessing schemes (box crop vs. random crop).

Figure 15 shows the validation results of the box and random crop implementation in SlowFast 8 × 8 ResNet-50. The accuracy of the random crop and box crop methods are approximately 80% and 85%, respectively; the box crop method showed higher accuracy. In the case of the random crop method, the recognition rate is relatively lower, and the learning process is less stable, as the abnormal state may not be adequately represented or may occupy a small portion of the cropped area. The result of the box crop shows the occurrence points of the outlier, in which the pixel error value increases in the reconstruction frame in the 3D-AE. It focuses on the crop, and the characteristics of the outlier state can be classified well.

### 5.4. Evaluation of the System Performance in Real World

**Figure 16:**
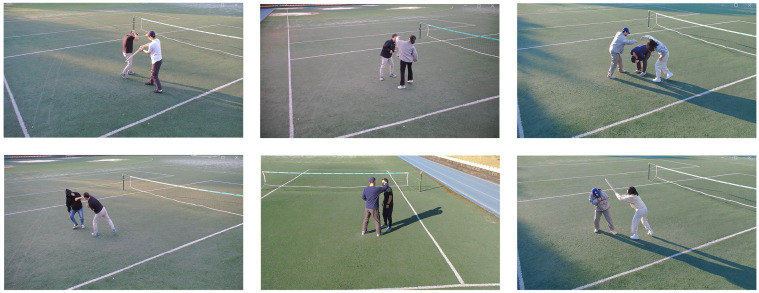
Assault data-constructed sample from real world.

**Figure 17:**
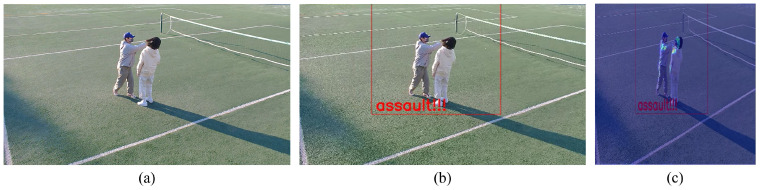
Proposed system test results: (**a**) input data, (**b**) classification result, and (**c**) classification result-masked VAD result.

Furthermore, we assessed the model’s performance using a dataset in a real-world environment to verify that the proposed system performs effectively in the real world. The generated data exclusively focused on the assault class, as it was the most straightforward class to reproduce among the learned classes. Furthermore, the dataset incorporates various viewpoints, assault methods (punch, kick, etc.), the number of individuals involved, and the attire of the objects to accurately depict real-world scenarios. We generated a total of 1345 assault data samples, and the sample images are presented in Figure 16. In the test of the GTA-trained multi-modal system (utilizing the box crop method) shown in Table 3, 1123 instances were classified as assault, resulting in a recall rate of 83.49%. Furthermore, we confirm that the detection results of VAD closely matched the actual location of the anomaly. As shown in (c) of Figure 17, the reconstruction error map of VAD was masked to the classification result in (b). The results confirmed that the VAD detection outcomes were consistent with the actual location of the anomaly.

## 6. Conclusions

In this paper, we propose an intelligent anomaly surveillance and security system based on multi-modal fusion using deep learning, aimed at achieving fully automated monitoring in security facilities. We have presented a method that combines VAD and VAC to overcome the limitations of the existing surveillance systems. Furthermore, by incorporating detection information from VAD during the fusion process, we substantially improved the classification accuracy of the VAC. In addition, we have fabricated a distinctive dataset using the GTA virtual environment. By utilizing a virtual environment that closely resembles the real world, it became feasible to generate a dataset comprising diverse threats and environmental elements that are challenging to replicate in reality. This approach addressed the challenge of applying existing open datasets to real-world scenarios, as these datasets often feature only simplistic backgrounds in the context of actual surveillance monitoring system applications.

In future research, we plan to conduct additional experiments involving the implementation of diverse data classes in the real-world environment. Our focus will be on developing a more extensive anomaly state and behavior detection and classification by applying a complex classification model that combines real-world and virtual-world data. In addition, by implementing a model lightening method that can be directly mounted on edge-based CCTVs, we will implement a model optimization method in actual edge devices.

## Figures and Tables

**Table 1:** AUC score for Avenue, ShanghaiTech, and GTA (our dataset).

Model	Dataset	AUC Score (%)
Skip-frame-based 3D AutoEncoder	Avenue	84.67
Skip-frame-based 3D AutoEncoder	ShanghaiTech	75.97
Skip-frame-based 3D AutoEncoder	GTA (ours)	79.95

**Table 2:** SlowFast test result for GTA (own dataset) and UCF-101 and HMDB.

Model	Dataset	Preprocessing	Accuracy (%)	Loss
SlowFast 4 × 16 ResNet-50	GTA (ours)	Box crop	78.75	0.54
SlowFast 4 × 16 ResNet-101	GTA (ours)	Box crop	75.00	0.67
SlowFast 8 × 8 ResNet-50	GTA (ours)	Box crop	85.00	0.53
SlowFast 8 × 8 ResNet-101	GTA (ours)	Box crop	80.00	0.57
SlowFast 8 × 8 ResNet-50	GTA (ours)	Random crop	77.50	0.71
SlowFast 8 × 8 ResNet-101	UCF-101	Random crop	72.31	1.02
SlowFast 8 × 8 ResNet-101	HMDB-101	Random crop	37.50	5.81

**Table 3:** Test result in the real-world assault dataset.

Data Amount	True Positive (TP)	Recall (%)
1345	1123	83.49

## Data Availability

Data is contained within the article.
